# A Novel Micro- and Nano-Scale Positioning Sensor Based on Radio Frequency Resonant Cavities

**DOI:** 10.3390/s140609615

**Published:** 2014-05-30

**Authors:** Estibaliz Asua, Victor Etxebarria, Alfredo García-Arribas, Jorge Feutchwanger, Joaquín Portilla, Julio Lucas

**Affiliations:** 1 Departamento de Electricidad y Electrónica, Universidad del País Vasco UPV/EHU B° Sarriena s/n Leioa 48940, Spain; E-Mails: victor.etxebarria@ehu.es (V.E.); alf@we.lc.ehu.es (A.G.-A.); jorge.feuchtwangerm@ehu.es (J.F.); joaquin.portilla@ehu.es (J.P.); 2 BCMaterials, Universidad del País Vasco UPV/EHU, B° Sarriena s/n Leioa 48940, Spain; 3 Elytt Energy, Paseo de la Castellana 114, 3°, 728046 Madrid, Spain; E-Mail: Julio.lucas@elytt.com

**Keywords:** positioning sensors, resonant cavities, precision metrology

## Abstract

In many micro- and nano-scale technological applications high sensitivity displacement sensors are needed, especially in ultraprecision metrology and manufacturing. In this work a new way of sensing displacement based on radio frequency resonant cavities is presented and experimentally demonstrated using a first laboratory prototype. The principle of operation of the new transducer is summarized and tested. Furthermore, an electronic interface that can be used together with the displacement transducer is designed and proved. It has been experimentally demonstrated that very high and linear sensitivity characteristic curves, in the range of some kHz/nm; are easily obtainable using this kind of transducer when it is combined with a laboratory network analyzer. In order to replace a network analyzer and provide a more affordable, self-contained, compact solution, an electronic interface has been designed, preserving as much as possible the excellent performance of the transducer, and turning it into a true standalone positioning sensor. The results obtained using the transducer together with a first prototype of the electronic interface built with cheap discrete elements show that positioning accuracies in the micrometer range are obtainable using this cost-effective solution. Better accuracies would also be attainable but using more involved and costly electronics interfaces.

## 1. Introduction

Micro- and nano-scale positioning measurement is becoming increasingly important in ultra- precision metrology and manufacturing, and in general, in any kind of micro- or nano-technological application. Among the many ways of sensing displacement [1,2], laser interferometry [3,4] and capacitive [5] and inductive sensors [6] are nowadays considered the most feasible solutions in nano-scale applications. However, several issues may limit the use of these kinds of sensors. Depending on the technology, these drawbacks are related to laser wavelength stability, volume and cost of optical components, fringe effects, proportionality errors, non-linearity or hysteresis, among others. Therefore, alternative complementary methods of measuring displacement at very small scales remains an open field of research of high technological interest. Many new variations of the above measuring principles, aimed at micro- and nano-scale applications are continuously being developed. This is the case, for instance, of the combination of linear encoders with arrays of micro-electrodes presented in [7], or the use of capacitive sensors in incremental mode to increase its resolution shown in [8]. Other novel solutions for the problem involve the use of new materials, such as magnetostrictive TbDyFe [9] or carbon nanotubes [10], for example. Finally, the use of radio frequency (RF) principles in displacement detection is also becoming increasingly important [11–13].

In this work, a new approach to subnanometric displacement measurement, based on a new working principle that uses radio frequency resonant cavities is presented. A prototype transducer design using two resonant cavities operating in differential mode has been constructed and tested in the laboratory [14]. It is shown that the frequency output of the experimental device changes by as much as several kHz per nanometer, meaning that subnanometric displacements can easily be detected and measured using this device by means of standard instrumentation. The transducer can be operated by discriminating resonant frequency variations as a function of displacement using an RF network analyzer (NA), but this approach limits the use of this kind of sensor in applications where the use of that instrumentation is not feasible, especially for its cost. Although high sensitivity displacement sensors usually require expensive signal conditioners, we also present here a cost-effective prototype of an electronic interface to be used together with the resonant cavity transducer in order to replace the NA with minimum loss of resolution and accuracy. The cavity equipped with the proposed electronic interface results in a complete standalone displacement sensor, giving a measurable output proportional to displacement or position.

The paper is organized as follows: in the next section the full sensor description and its operating principle are described. Then, in the third section, the experimental laboratory prototype is presented. The next section shows the experimental results obtained with the prototype and finally, some conclusions and future works are given in last section.

## 2. Principle of Operation of the Sensor

### 2.1. Transducer Description and Principle of Operation

**Figure 1.**
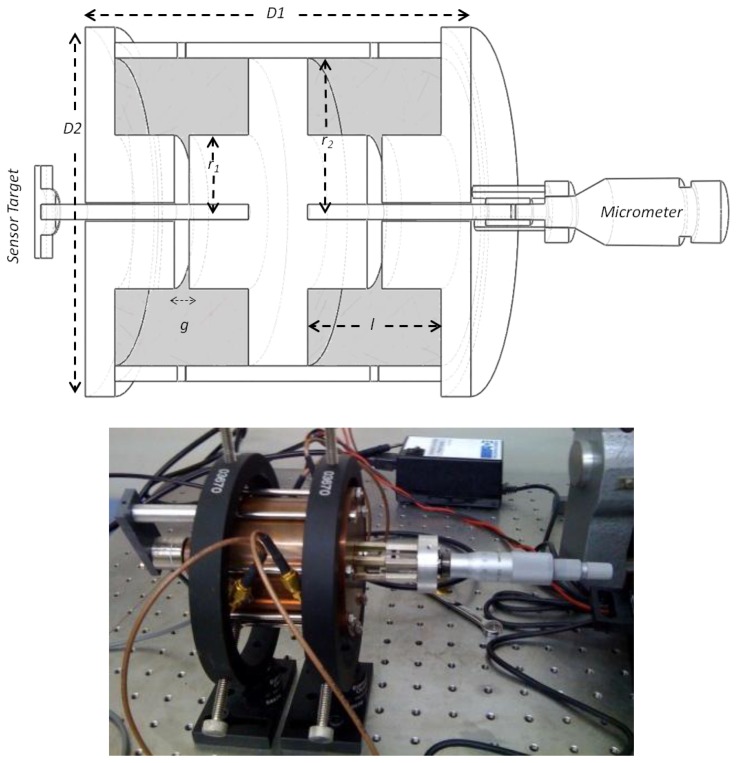
General scheme and photograph of the laboratory setup for testing the cylindrical displacement sensor prototype based on resonant cavities. A section-cut of the sensor is shown in the three-dimensional scheme. The white part constitutes the body of the sensor. The movement or position to be measured is transmitted to the central part through the rod. The parts in grey constitute the cavities. The movement of the central part changes the resonant frequency of each cavity in differential form. For the laboratory tests, the rod displacements are generated with an external micrometer, and measured with and external capacitive sensor.

The proposed sensor design measures displacement by reading electromagnetic variations inside resonant cavities of changing geometry. Figure 1 shows a general scheme of the laboratory prototype sensor made of copper comprising two resonant cavities whose dimensions change in opposite directions as a function of the displacement of a transmission rod. The cavities are arranged to optimize their sensitivity to the displacement of the rod, so that even for tiny geometry changes in the cavities, the resulting signal variations can be easily measurable in the resonant circuit.

As shown in Figure 1, a 60 mm diameter prototype resonant cavity of the re-entrant type is used in the laboratory setup. Three fundamental parts that are drawn in white constitute the body of the transducer. The central block is moved by the transmission rod, and its movement entails a dimension variation in the resonant cavities (represented in grey), which are fed by RF coupling loops. In the cavities, the electrical field is concentrated on a small gap near the axis, while the magnetic field is mostly on the outer diameter. The reason to choose this type of cavity is the strong variation of the equivalent capacitance with the gap dimension. Let *g* be the gap, *r_1_* the radius of the gap region, *r_2_* the outer radius of the cavity and *l* its length. Then the resonant frequency, *f*, at the central position is:

(1) $$f=\frac{c\sqrt{2g}}{2\pi r_{1}\sqrt{l\ln(r_{2}/r_{1})}}$$

where *c* is the speed of light, and the remaining parameters correspond to the characteristics of the cavity’s geometry and are shown in Figure 1. Those parameters determine the resonant frequency and the measurement range.

The absolute variation of frequency with the displacement, ∆*f*, may be found by taking a logarithmic derivative of the central frequency expression:

(2) $$\frac{\Delta f}{f}=\frac{1}{2}(\frac{\Delta g}{g}-\frac{\Delta l}{l})$$

In order to have a high sensitivity, the *g* gap dimension will be much smaller than the total cavity length, and therefore, the first term inside the parentheses dominates. It is very clear that a small gap is required to obtain a high resolution.

### 2.2. Principle of Operation of the Electronic Interface

Initially, the frequency response of both cavities was registered using a radio frequency network analyzer (model E8358A PNA, Agilent, Palo Alto, CA, USA). This was done to measure with high precision the remarkable characteristic curves (displacement versus resonant frequency change) of the transducer. However, an alternative way to interface the transducer has also been studied in order to replace a network analyzer and provide a complete practical sensor (transducer plus electronics) in an affordable, self-contained and compact solution. The first approach is based on developing a low cost electronic interface built using discrete typical RF components. The network analyzer simultaneously excites the cavity and measures its resonant frequency, so we divide the proposed electronic interface into two stages: the excitation part and the detection one.

#### 2.2.1. Excitation Stage

**Figure 2.**
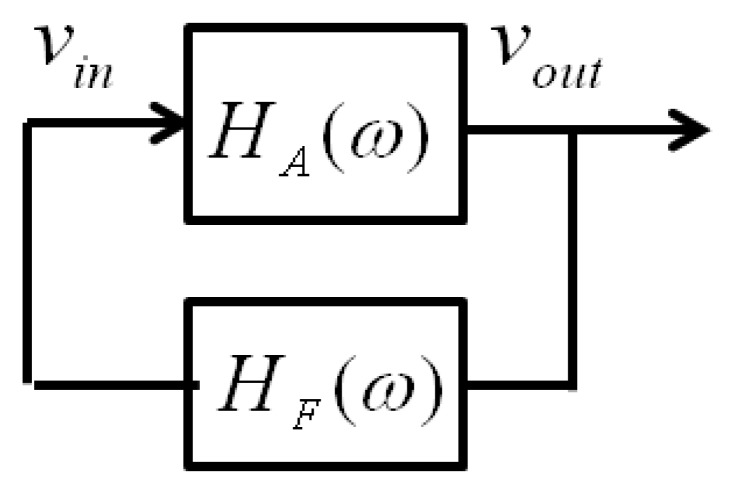
Scheme of the feedback loop to produce the self-oscillation of the resonant cavity. *H_A_*(*ω*) is a low noise amplifier. *H_F_*(*ω*) comprises the cavity and a phase shifter (see text).

The purpose of this part is to excite and induce oscillation in the resonant cavity. We have designed a feedback harmonic oscillator using the resonant cavity itself. A loop that causes a positive feedback at a selected frequency is the core of any oscillator circuit [15]. The transfer function of the loop shown in Figure 2 is described by:

(3) $$\frac{V_{\text{out}}}{V_{in}}=\frac{H_{A}(\omega )}{1-H_{F}(\omega )H_{A}(\omega )}$$

Since there is no input (V_in_ = 0), to obtain a nonzero output voltage, a Barkhausen criterion has to be satisfied, that is, the denominator in (3) has to be zero. Thus, these two conditions have to be satisfied:

(4) $$\begin{array}{l} |H_{F}(\omega )H_{A}(\omega )|=1\\ \arg[H_{F}(\omega )H_{A}(\omega )]=180{}^{\circ} \end{array}$$

In the proposed prototype, the *H_A_*(*ω*) is a low noise amplifier, whereas the feedback system *H_F_*(*ω*) is the cavity itself. The amplifier together with the cavity satisfies the first condition, but to meet the second requirement, it is necessary to introduce a phase shifter in the feedback in order to add the necessary phase to the system to produce the self-oscillation of the device.

#### 2.2.2. Frequency Detection

Once the cavity resonates, its output frequency (which is related to the position) has to be measured. To do so, a low pass filter and two RF power detectors are used. The low pass filter has been designed to attenuate the signal depending on its frequency. The first RF power measures the signal before passing through the low pass filter. The second one measures the signal once it has been attenuated. The ratio between those two measurements, that is, the attenuation of the signal, gives us the resonant frequency of the cavity.

## 3. Experimental Prototype

**Figure 3.**
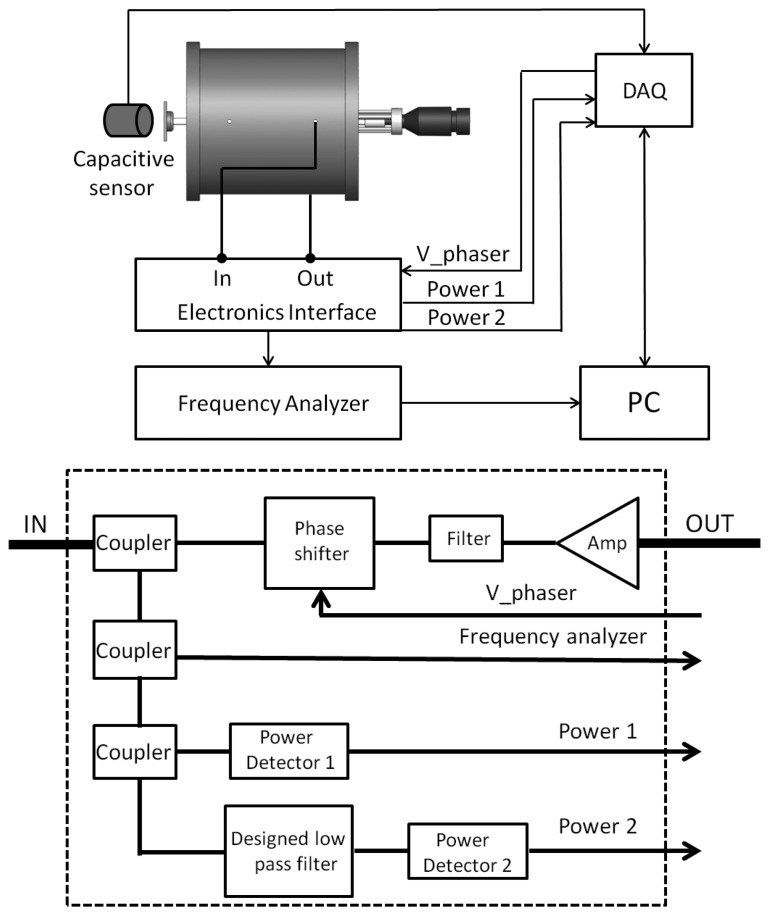
Scheme of the positioning sensor prototype. The complete device is described above, whereas electronic interface built with RF discrete elements is shown below.

Figure 3 displays the experimental setup that has been used to test and calibrate the prototype sensor. The complete device was fixed to a vibration isolation table using standard optical fixtures (Edmund Optics Ltd., Pennsbourg, PA, USA).

The transmission rod of the transducer is displaced in a controlled way by means of a micrometer head (model 153–201, Mitutoyo, Kawasaki, Japan) attached to it. The displacement is measured with a high performance capacitive sensor (model 4810, ADE Technologies, Lowell, MA, USA) attached to the rod opposite to the micrometer.

In the first experiments the transducer is used together with the network analyzer but, once the transducer’s sensitivity is tested, the electronic interface built using discrete elements has been added to complete the system as Figure 3 displays. The frequency analyzer is also included to check the electronic interface performance.

As it has been explained before, the cavity together with a low noise amplifier (ZFL-1000LN, MiniCircuits, Brooklyn, NY, USA) and a phase shifter (JSPH-661) form the feedback harmonic oscillator. A filter (VLF-630, MiniCircuits, Brooklyn, NY, USA) is included after the amplifier to clean its output. The phase shifter is controlled by a voltage (*V_phaser*) that varies between 0 and 10 V in order to shift the phase between 0 and 270 degrees. In the detection stage, the signal produced by the oscillator is fed into the first power detector (ZX47-50, MiniCircuits, Brooklyn, NY, USA) and the low pass filter. Once the signal is attenuated by this filter, its power is measured again, using the second power detector (ZX47-50). The measurements from the power detectors and the *V_phaser* signal are managed using a data acquisition board (Model 6011, National Instruments, Austin, TX, USA) and LabVIEW software. To determine the accuracy of the electronic interface, the signal produced by the resonant cavity is simultaneously measured by a frequency analyzer.

Three ZX30-12-4-S+ couplers have been added when needed to route signals. They produce some signal attenuation, but this attenuation is independent of the frequency, so, they do not change the proposed methodology.

## 4. Experimental Results

### 4.1. Transducer Sensitivity

As it has been mentioned before, in order to test the transducer response, the frequency response of both cavities has been registered using a radio frequency network analyzer (Agilent model E8358A PNA) and the resonant peak frequency for each cavity was measured along the whole displacement range (400 μm) of the transmission rod. For the specific geometry of the prototype, each individual cavity has been measured to change its resonant frequency from 275 MHz to 750 MHz approximately.

**Figure 4.**
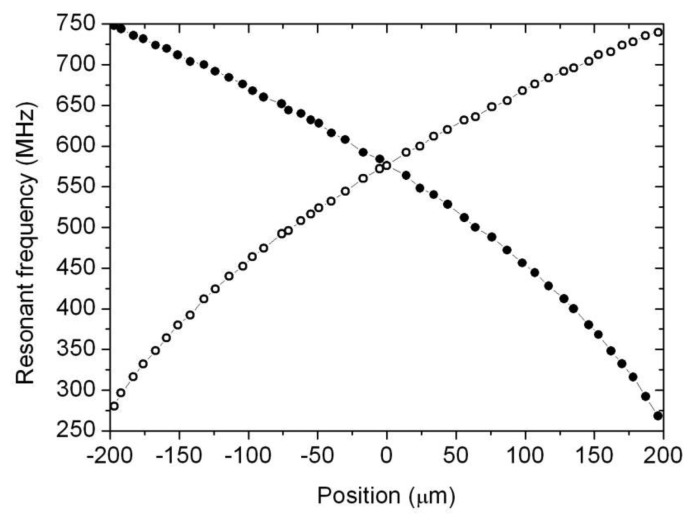
Change in the resonant frequency in both cavities as a function of the displacement. Since both cavities are mechanically coupled by the central piston, any displacement will always result in opposite frequency shifts.

In Figure 4 the resonant frequency values in the complete displacement range of the prototype measured at intervals of roughly 10 μm are depicted. The external capacitive sensor readings are represented in the horizontal axis, while the vertical axis displays the resonant frequency for each position for both cavities. As it can be observed, since both cavities are mechanically coupled by the centre piston, any displacement will always result in the resonant frequency of one cavity increasing and that of the other one decreasing. The relation between resonant frequency and position in each cavity is almost linear in small displacement ranges, and could be represented by a second order polynomial over the whole tested range.

**Figure 5.**
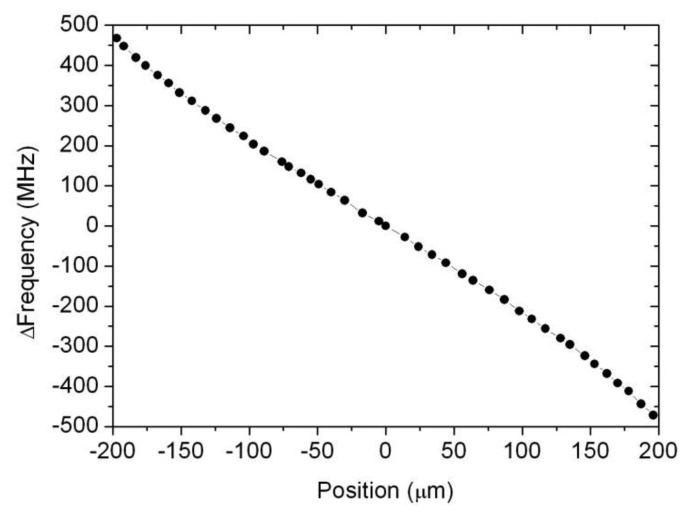
Change of frequency in sensor output as a function of displacement. Two cavities in differential mode are represented in vertical axis. An excellent sensitivity of 2.25 kHz/nm is obtained.

Although one cavity would be enough to sense displacement, the use of the two cavities in differential mode is proposed here to enhance the transducer’s performance. In Figure 5 the vertical axis displays the difference of peak resonant frequencies between both cavities for the same positions shown in Figure 4. As it can be observed, using two cavities in differential mode doubles the sensitivity of the device and compensate slight nonlinearities and possible inaccuracies due to thermal expansion of the cavities. It also compensates the slight parabolic behaviour in the data corresponding to each individual cavity which was observed in Figure 4. As shown in Figure 5, the experimental points (difference of frequencies between both cavities versus displacement) nicely fit now in a linear relation between displacement and frequency. Moreover, it is remarkable the very high sensitivity of the device, whose response changes as much as 2.25 MHz in frequency for each micrometer. This makes an excellent sensitivity of 2.25 kHz/nm.

### 4.2. Excitation Stage

**Figure 6.**
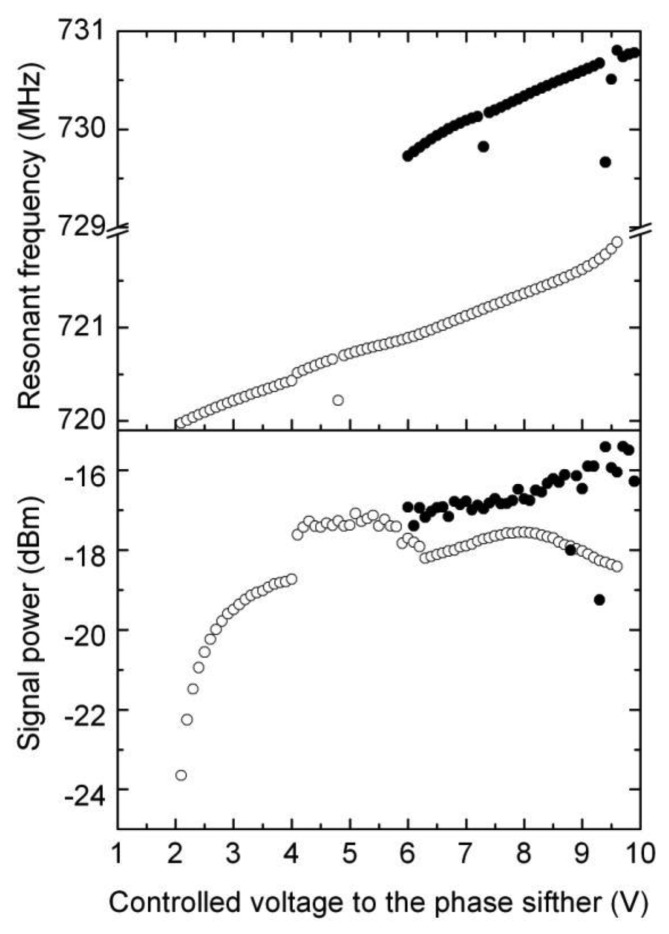
Resonant frequency (measured using a frequency analyser) and produced power (measured with the power detector 1, Figure 3) during the self-oscillation of the cavity. The self-oscillation is produced for different values of the introduced phase shift, which is represented in the *x*-axis of the figure by the voltage applied to the phase shifter. The data is represented for two positions of the sensor: 160 μm in empty circles and 170 μm in filled circles.

**Figure 7.**
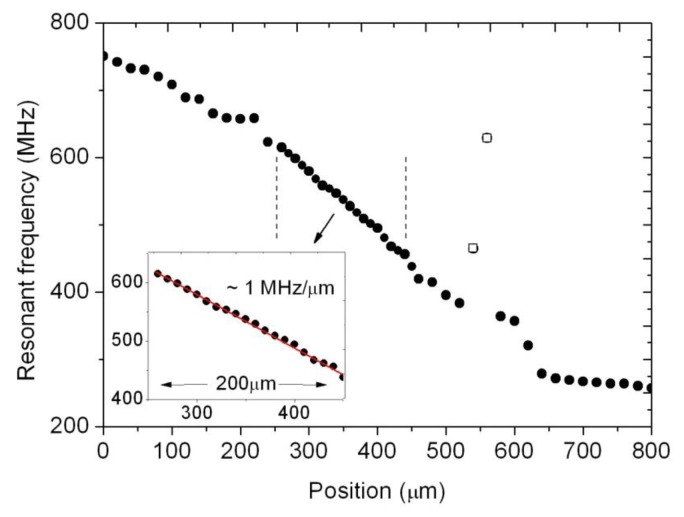
Self-oscillation frequency of the cavity as a function of position. The phase selected to produce the oscillation is the one delivering the maximum power for each position. The inset highlights the linear range selected for operation.

This part of the electronic interface implements the proposed feedback harmonic oscillator, so that oscillation is induced in the resonant cavity by introducing an extra phase in the loop shown in Figure 2, by means of a phase shifter as explained above. The measurements reveal that the method works as expected, although we have observed that for a given position, the cavity oscillates for different values of the phase introduced by the phase shifter. Moreover, for a given position of the resonant cavities (that is, for a given position to be measured by the sensor), the power of the obtained signal and even the resonant frequency depend on the actual value of the phase (see Figure 6). In this situation, we decided to calibrate the sensor by selecting the phase delivering the maximum power for each position (see Figure 7). This resulted in a linear useful range of 200 μm (noted in the figure) for the sensor.

### 4.3. Low Pass Filter Calibration

**Figure 8.**
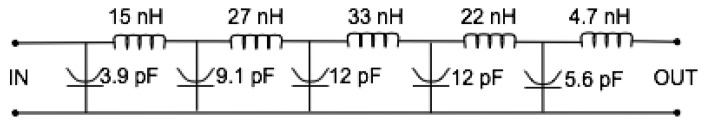
Elements comprising the low pass filter.

**Figure 9.**
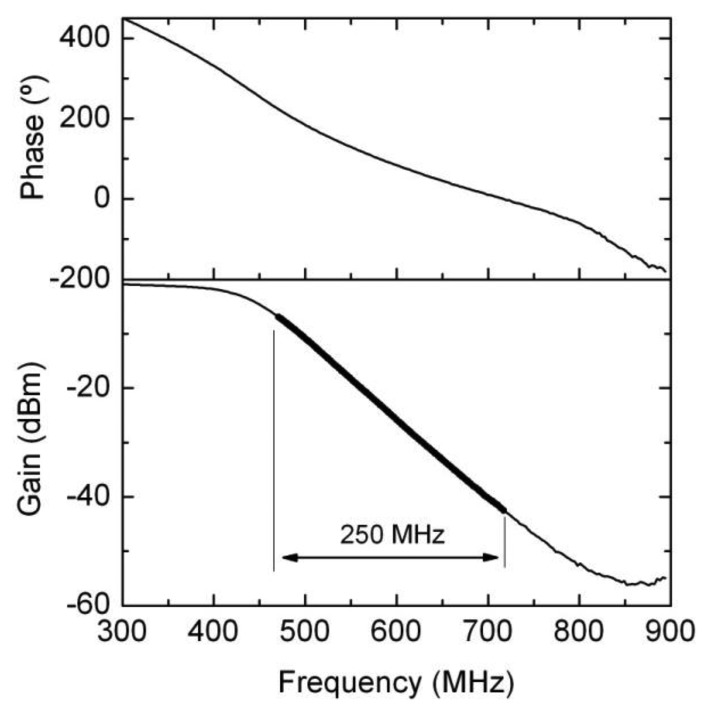
Low pass filter performance. Gain and phase of the filter measured using a network analyzer.

As it has been previously explained, a low pass filter has been designed in order to attenuate the signal depending on its frequency. Figure 8 shows the elements comprising the filter, and in Figure 9 its performance as measured with a frequency network analyser is displayed. As shown, the filter’s response is linear in the working frequencies.

**Figure 10.**
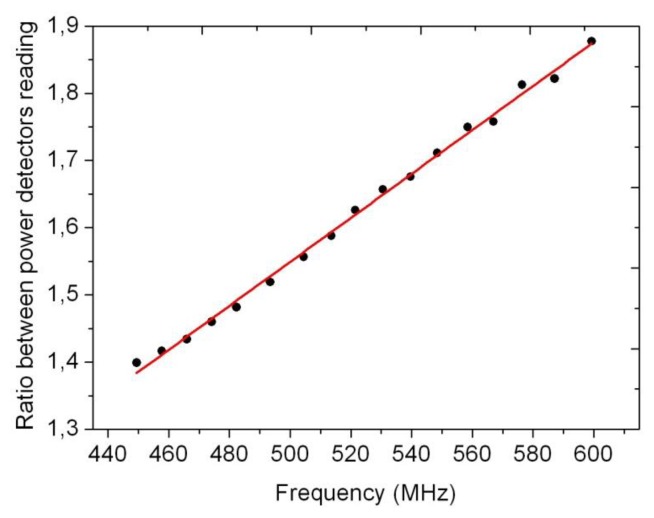
Ratio between the power detector readings. It represents the filter’s response depending on the frequency of the self-oscillation of the cavity.

As it has been already mentioned, the couplers introduced to route the resonant signal to the different electronic elements (see Figure 3) also produce some attenuation. In order to take account for it, the calibration has been done using the ratio between the second power detector to the first one when they are connected as the diagram of Figure 3 indicates. Figure 10 displays the results that are conveniently fitted to a linear expression:

(5) $$\text{Ratio}=0.00327f_{\text{r}}(\text{MHz})-0.0858\left(r=0.996\right)$$

**Figure 11.**
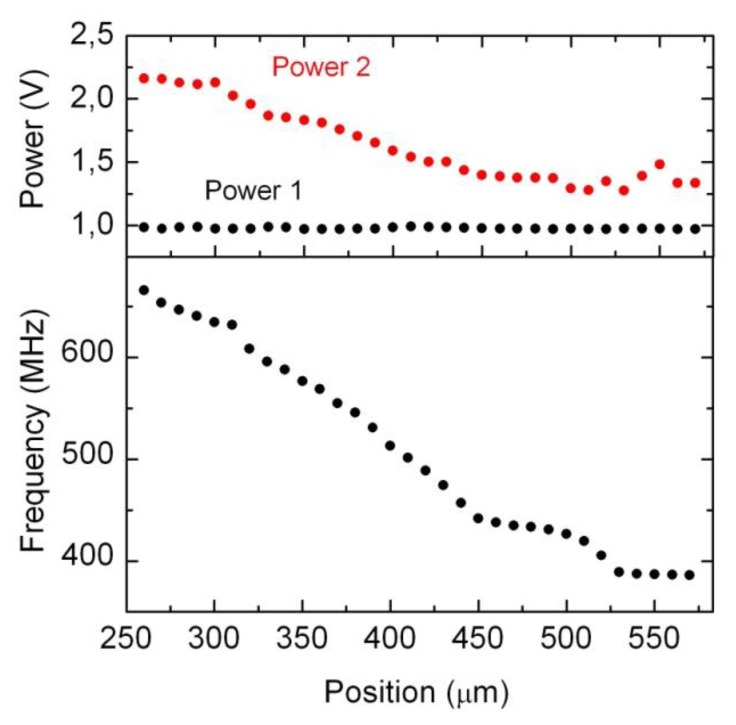
Experimental results. On top, the voltage measured by the power detectors. Below, the calculated frequency based on the ratio of powers and calibration (Equation (5)).

Thus, the method to determine the resonant frequency consists of measuring the power of the signal before and after it passes through the filter and using Equation (5) to deduce the frequency. Two nested loops are implemented: the first “coarse” one sweeps the phase shift to achieve oscillation, and the second “fine” one sweeps around this position to reach the prefixed allowable (0.97–0.99 V) power reading. Once this is achieved, the resonant frequency is determined from both power detector readings. In Figure 11 the voltages in the first and second power detectors, as well as the calculated frequency based on the ratio between powers and the calibration (Equation (5)) are shown.

**Figure 12.**
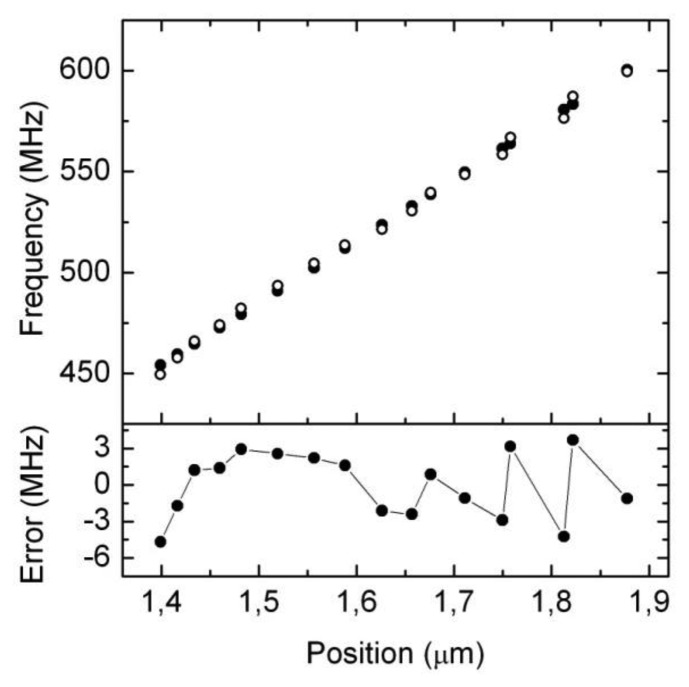
Experimental results. On top, resonant frequency as given by the network analyzer (full circles) and by the proposed electronic interface (open circles). Below, difference between them (that is, the error when using the electronic interface).

In order to check the performance of the system we compare the frequency as measured with a laboratory calibrated network analyzer and the one given by the proposed electronic interface. As it can be concluded observing Figure 12, the difference between both readings is about 5 MHz, which corresponds to 5 micrometers according to the single-cavity transducer intrinsic sensitivity as explained in Section 2 (Figure 4). This error could be improved if we restricted the measurement range of the sensor, or even better if we further constrained the allowable power readings in the first power detector. This latter approach would require a longer time to stabilize the second “fine” loop described above, so there is a clear trade-off between measurement time, precision and range. In any case, from the above experimental results, it can be concluded that the proposed electronics interface is a cost-effective, yet accurate solution for the proposed new displacement and position transducer based on radio frequency resonant cavities.

## 5. Conclusions/Outlook

We have developed and tested a novel position and displacement transducer based on radio frequency resonant cavities, whose response is linear and exhibits very high sensitivity (higher than 1 MHz per micrometer) in laboratory tests. These experimental results have demonstrated the practical feasibility of this new way of sensing displacement for micro and nanometric applications.

In order to use the transducer as a standalone sensor, that is, in order to obtain a measurable voltage proportional to the position without the need of costly instrumentation such as network analyzers, a first electronic interface prototype based on cheap and common RF discrete elements (low pass filters, power detectors and a low noise amplifier) has been designed and tested. It has been shown that accuracies around 5 MHz in frequency (which corresponds to less than 5 micrometers in displacement) are achieved using this interface. This performance could be further improved restricting the range or increasing the measurement time.

As a general summarized conclusion, it can be stated that the designed displacement transducer based on resonant cavities together with the first developed prototype of the cost-effective electronic interface, constitute a new complete standalone sensor useful for measuring displacements in the micrometer range. Alternative interface approaches are also being considered at present to take better advantage of the high sensitivity of the transducer. One of those alternatives would be based on using the RF cavity to implement a variable low pass filter, in such a way that the displacement to be measured produced changes in the attenuation of the filter. Then, a fixed frequency oscillator could be used to supply a fixed amplitude RF signal, and the magnitude of the signal after the filter would give the position measurement. This general approach has been successfully used in sensors based on self-inductance changes [16]. Other approaches using more involved and costly electronics interfaces are also being considered at present to push the presented displacement transducer to its limits.
